# Estimating ground-level PM_10_ using satellite remote sensing and ground-based meteorological measurements over Tehran

**DOI:** 10.1186/s40201-014-0122-6

**Published:** 2014-09-07

**Authors:** Saeed Sotoudeheian, Mohammad Arhami

**Affiliations:** Department of Civil Engineering, Sharif University of Technology, Azadi Ave, P.O. Box 11155–9313, Tehran, Iran

**Keywords:** PM_10_, Particulate matter, Remote sensing, Aerosol optical depth, AOD, MODIS, MISR, Multivariable regression models

## Abstract

**Background and methodology:**

Measurements by satellite remote sensing were combined with ground-based meteorological measurements to estimate ground-level PM_10_. Aerosol optical depth (AOD) by both MODIS and MISR were utilized to develop several statistical models including linear and non-linear multi-regression models. These models were examined for estimating PM_10_ measured at the air quality stations in Tehran, Iran, during 2009–2010. Significant issues are associated with airborne particulate matter in this city. Moreover, the performances of the constructed models during the Middle Eastern dust intrusions were examined.

**Results:**

In general, non-linear multi-regression models outperformed the linear models. The developed models using MISR AOD generally resulted in better estimate of ground-level PM_10_ compared to models using MODIS AOD. Consequently, among all the constructed models, results of non-linear multi-regression models utilizing MISR AOD acquired the highest correlation with ground level measurements (R^2^ of up to 0.55). The possibility of developing a single model over all the stations was examined. As expected, the results were depreciated, while nonlinear MISR model repeatedly showed the best performance being able to explain up to 38% of the PM_10_ variability.

**Conclusions:**

Generally, the models didn’t competently reflect wide temporal concentration variations, particularly due to the elevated levels during the dust episodes. Overall, using non-linear multi-regression model incorporating both remote sensing and ground-based meteorological measurements showed a rather optimistic prospective in estimating ground-level PM for the studied area. However, more studies by applying other statistical models and utilizing more parameters are required to increase the model accuracies.

## Background

Increasing levels of air pollutants has become a complex issue affecting public health and environment in various cities of the developing countries during the recent years [1]. Serious adverse health effects such as respiratory problems, cardiovascular and lung disease and other damaging effects on human health has been associated to the air pollutants [2-5]. Among different pollutants, particulate matter (PM), including PM_10_ and PM_2.5_ (PM with aerodynamic diameters less than 10 μm and 2.5 μm, respectively), raised thoughtful concerns regarding public health [6-10]. In order to effectively manage the pollutants and evaluate the efficiency of different control strategies, it is crucial to determine the pollutant levels and their variations in different environments [11] which is generally done through air pollution monitoring networks.

Although the ground-level measurements are generally referred to as accurate methods, these measurements indicate the pollution concentrations of a small area around the monitoring stations. Consequently, the studies of air pollutants and their adverse effects are impeded by limited coverage and irregular distribution of monitoring stations at ground level [12]. In fact, achieving comprehensive pollutants coverage from ground based measurements is difficult due to the limited number of stations equipped with costly instruments [13]. Hence, researchers have been constantly seeking new methods to attain more comprehensive measurements.

In the past decades, innovations in the field of remote sensing techniques by satellites opened a new era for different measurements including air pollutants measurements. Particular attempts have been made in the satellites based remote sensing of PM concentration in the lower troposphere since the late 1970s. Several sensors measure the parameters associated to concentration of aerosol in the atmosphere [14].

The Moderate Resolution Imaging Spectroradiometer (MODIS) sensor on Terra and Aqua satellite and Multiangle Imaging SpectroRadiometer (MISR) on Terra sensors measure the particle abundance and their composition by determining Aerosol Optical Depth (AOD) with temperate spatial resolutions [15]. AOD which reflects optical characteristics of aerosols is also determined by other sensors such as Ozone Monitoring Instrument (OMI) on Aura and is measured by sunphotometer in the Aerosol Robotic Network (AERONET) network. The AOD measurements have been used in several studies to estimate PM_2.5_ or PM_10_ concentration at the ground level [16].

Using the measurements by satellite sensors to estimate ground-level particulate concentration is a challenging endeavor. Several factors such as particle composition and physical properties affect the optical properties of particles and consequently influence the relationship between satellite data and PM_10_ concentration. Also upper air obstacles including dense cloud cover, and variations in vertical PM profile could disturb this relation, or lead to missed data in the pixels of interest. Hence, it is crucial to integrate other variables and build a model to estimate ground based particulate levels. In these models satellite based measurements are combined with other variables and compared to concentration data to estimate PM concentration and provide valuable information to establish effective air quality strategies and high accuracy predicting models. Two approaches have been implemented in modeling process to estimate PM_10_ concentration. First approach is utilizing the deterministic models requiring intensive data including the inventory of pollution sources, which can be difficult to quantify. Second approach, which is the focus of our study, utilizes statistical models to optimize the relationship between PM levels and independent variable [12].

Several studies have been conducted to develop a relationship between satellite AOD data and PM concentration at the ground surface. Examples of these studies, which were mainly carried to obtain reliable estimates of PM concentration, are presented in the following. Wang et al. [17] examined linear relationship between hourly PM_2.5_ concentration and AOD from MODIS at 7 stations in Jefferson County, Alabama (R = 0.7). The rather high correlation was found between monthly average of PM_2.5_ and AOD (R > 0.9) [17]. Some other similar works obtained relationships between PM_2.5_ and PM_10_ concentration and AOD with R^2^ range of 0.58 to 0.76 [18-20].

By advancing studies in the field of using remote sensing to estimate PM concentration, the structures of models changed from simple linear models to the more complex non-linear models by using the other affecting parameters such as meteorological data. In this regard, Liu et al. [21] generate empirical model to estimate PM_2.5_ concentration at surface level using the AOD data from MISR sensor. Results show that their model can explain 48% of the variability in PM_2.5_ concentration. They found that several factors such as relative humidity, planetary boundary layer height, season and geographical characteristics of area can affect the relationship between PM_2.5_ and AOD data [21]. Liu et al. [15] continued their studies in 2007 and developed, two general linear regression models by using AOD from both MODIS and MISR sensors and compared their performances. The MISR model was able to explain 62% of variability in PM_2.5_ concentration, while MODIS model explained 51% of concentrations variability [15]. Vladutescu et al. [22] showed that incorporating variation of planetary boundary layer height in such models can improve the accuracy of the models. Also, they showed physical characteristics and hygroscopic of particles (relative humidity of atmosphere) have significant effect on models performance [22]. In the similar work, Koelemeijer et al. [23] advanced PM-AOD relationship by incorporating effect of several factors such as boundary layer height and relative humidity on particles size. Average correlation coefficients between measured and modeled levels were 0.5 and 0.6 for PM_10_ and PM_2.5_, respectively in rural and suburban regions [23]. Pelletier et al. [24] also improved the performance of linear model between AOD and PM concentration by adding auxiliary parameters, mainly meteorological variables. Their improved linear model could explain 76% of concentrations variability [24]. Vidot et al. [25] continued Pelletier et al. [24] general idea, and amended PM and AOD (from SeaWiFS imagery) relationship with effective meteorological information through a statistical approach. They obtained determination coefficient of 0.42 and 0.48 for PM10 and PM2.5 respectively [25]. Also, Gupta et al. [26] developed multiple regression models between meteorological parameters and AOD data from MODIS sensor. Results show that significant improvement in correlation coefficients which was obtained by multiple regression tool and increasing meteorological parameters number [26]. In a more recent study, Tian et al. [12] generated a semi-empirical model in the regional scale to estimate hourly PM_2.5_ concentration. This model utilizes a modified AOD value based on boundary layer height and meteorological characteristics, which resulted in explaining 65% of PM_2.5_ variability [12]. Subsequently, Tian et al. [27] emphasized that the spatial and temporal variation of relationship between AOD and PM_2.5_ isn’t clear so far due to: meteorological condition, land use, cloud contamination, station location, and particle size. Majority of these recent investigations were able to build models to estimate PM_2.5_ concentrations rather than PM_10_.

These studies concurred on the capability of utilizing remote sensing as a powerful tool in predicting PM concentration at the ground surface especially for areas without a monitoring network. However, the results of most of these studies prove the need for more studies to increase the accuracy and reliability of the models by incorporating optimal parameters. The models and estimations accuracy was shown to vary for different regions of the world. Moreover, the ability of models to estimate high PM levels due to different phenomena such as dust storms, which could result in wide range of PM levels, has not been evaluated.

Tehran is one of the most polluted cities in the world, facing major issues raised by airborne particulate matter [28]. Many factors including growing populations, extensive transportation network, industrial emissions, and dust storm from deserts in neighboring countries such as Iraq and Saudi Arabia, affect the air quality situation in this metropolis. High PM levels also occasionally occur in this city’s atmosphere due to different phenomena such as dust storms, which results in wide range of PM levels. Despite the significance of the airborne particulate problem and possibility of using AOD data to predict surface PM_10_ concentration, no such research has been carried out in this region.

In this study, a semi-empirical equation was developed and examined to estimate the PM_10_ concentration in Tehran’s stations by utilizing the AOD data from MODIS and MISR sensors. Initially, individual models were developed, validated and evaluated for each station. Subsequently, general models for the entire region were built and examined. Due to the lack of such research in this region, results of this study could help the future investigations, and strategy developments to control airborne PM.

## Methods

### Data extraction and processing

**Figure 1:**
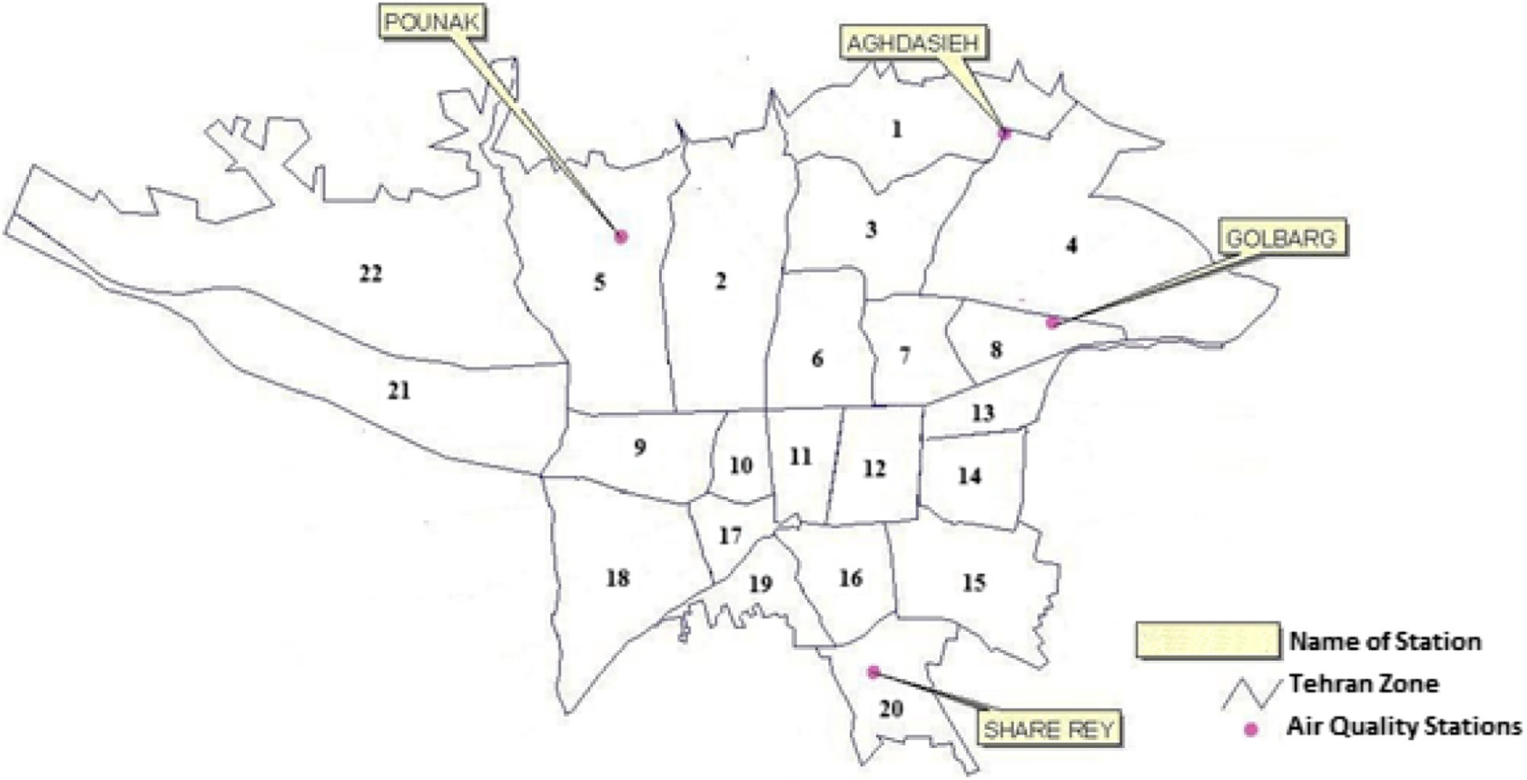
**Location of monitoring stations in Tehran.** The numbers in the figure represent the different districts of the city.

Tehran, capital of Iran, is a populated megacity extended from 51.2°E to 51.6°E, and from 35.4°N to 35.8°N. PM_10_ concentrations recorded at 4 stations from the air quality station networks operated by the Air Quality Control Company (AQCC) were used to calibrate and verify the models. Locations of the stations are shown in Figure 1. Hourly PM_10_ data measured continuously by Tapered Element Oscillating Microbalance (TEOM) instrument throughout the year 2009 were extracted for the means of this study. AOD data were extracted from the Moderate Resolution Imaging Spectroradiometer (MODIS) and Multi-angle Imaging SpectroRadiometer (MISR) sensors, and used in separate models. AOD parameter is obtained by integrating light extinction coefficient of pollutant in the atmosphere column from the ground level to the top by following equation (Equation 1):

1 $$ AOD={\displaystyle {\int}_0^{\infty }}{\sigma}_{ext}(h) dh $$

Where *σ*_*ext*_ is light extinction coefficient of pollutant at the height of H. *σ*_*ext*_ is calculated by Equation 2:

2 $$ {\sigma}_{ext}(h)={\displaystyle {\int}_0^{2.5\mu m}}{C}_{ext}\left(r,m\right)n(r) dr $$

where *C*_*ext*_ is extinction cross-sectional area, which is a function of particle size and refractive index, m, and size distribution of particle, *n*(*r*) [21].

MODIS sensors are installed on Terra and Aqua satellite platforms designed to retrieve aerosol properties over land and ocean [14]. These sensors collect data in 36 channels every 1–2 days depending on the data location [29]. This temporal resolution of AOD data make them appropriate for air quality assessments [14]. MODIS sensors with a 2330 km viewing swath provide almost complete global coverage in one day [30]. The MODIS AOD data are derived at three wavelengths of 0.47, 0.66, and 2.1 μm via over land retrieval algorithm. Total AOD at 0.55 μm is calculated by solving an inversion problem using independent observations of spectral reflectance data derived in three wavelengths (0.47, 0.66 and 2.1 μm) [31,32].

In this research AOD calculated at 0.55 *μm* from the Level-2 of MODIS (collection 5), which reflects the concentration of pollutant, is utilized due to its better quality and higher resolution (~10 km spatial resolution). The estimated uncertainty of MODIS data for the level 2 over land is 0.05 ± 0.15*AOD* [32]. The MODIS sensors onboard the Terra and Aqua satellite overpass Tehran at approximately 11 a.m. and 13:30 p.m., local time, respectively. After evaluating the data obtained on Terra and Aqua satellite, AOD data recorded by MODIS on Aqua in 2009 and 2010 were utilized due to the appropriateness and frequency of the circuit with Iran’s local stations (http://ladsweb.nascom.nasa.gov/).

MISR sensor is installed on Terra platform to study climate and environmental condition on a global scale. This sensor can provide images in nine view angles at four wavelength bands to retrieve aerosol information over land. MISR AOD data are recorded at a 17.6 km resolution with a temporal resolution of 2 to 9 days depending on the latitudes [15]. The AOD data by MISR which are obtained at 10:30–11:30 a.m. of Tehran’s local time due to the Terra platform orbiting schedule, were extracted for year 2009 and 2010 for the means of this study (http://eosweb.larc.nasa.gov).

The hourly ground-level PM_10_ concentrations used for modeling were extracted for the time at which the satellite data are recorded (12 a.m. and 1 p.m. for MODIS & 10 a.m. and 11 a.m. for MISR). Average PM_10_ levels over these time spans were utilized in the models. Once the models were calibrated using the processed data throughout 2009, data for the first half of 2010 were used to validate the models.

Size distribution, particle composition and vertical profile of aerosol are important factors affecting the relationship between satellite data and PM_10_ concentration at the ground level. Therefore, these factors should be considered in the models by implementing appropriate and plausible variables [21]. Meteorological parameters such as wind speed, wind direction, temperature and relative humidity have significant effects on PM_10_ concentration and particle optical properties. Particle properties can affect the relationship between PM_10_ concentration and corresponding remotely sensed data. For example, changes in relative humidity (RH) or temperature can directly or indirectly alter the particles composition due to change in photochemical oxidation and condensation processes, which affect optical properties of particles.

Also, change in aerosol size distribution and optical and physiochemical properties of aerosols could be occur due to generation of fresh particles from various sources and change in properties of particles in the atmosphere. Different factors such as photochemistry phenomena and hygroscopic growth are the main reasons of these variations in the atmosphere. Therefore, remote sensing parameters such as AOD, Angstrom exponent, single scattering albedo are also affected by such properties. Since the effect of pollutants characteristics affecting optical properties were not considered directly in the models, meteorological parameters were incorporated in the models as surrogates which indirectly reflect such effects. Temperature, and wind speed and direction were extracted from data recorded every 3 hours at Mehrabad synoptic station in Tehran for the purpose of this analysis. These data were extracted for 12:00 p.m. which was the closest to the time of recording AOD data by each sensor. Wind direction which plays an important role in particulate concentration was incorporated in the model using Dir parameter from the following equation (Equation 3) to include its rotational properties of having same identity at 0 and 360 degree (it should be noted that south and north wind showed rather similar effects):

3 $$ Dir= \cos \left(\frac{2\pi \theta}{360}\right) $$

$$ \theta = Wind\  Direction $$

Since the RH values could change particles composition and optical properties, this parameter was also incorporated in the models to improve their ability to estimates the PM_10_ concentration. Since hourly value of RH data weren’t available for the studied period, daily value of RH reported from synoptic station were used. Another implemented parameter was Planetary Boundary Layer’s Height (PBLH) which is the depth of the surface layer of the atmosphere. The fluid dynamic properties of this layer are directly influenced by contacting the planetary surface, and its height plays an important role in pollutants behavior in the atmosphere. In this study the PBLH were extracted from the Global Data Assimilation System (GDAS) files (http://www.ncdc.noaa.gov/). Spatial resolution of these data is 1^*°*^ × 1^*°*^ with a temporal resolution of 3 hours. PBLH data at 12:00 p.m. were used, which was the closest time to the AOD data recording time.

Quality Assurance and Quality Control (QA/QC) was performed on AOD and PM_10_ dataset. Initially by extracting the average and standard deviation (σ) of data, the data out of -3σ to 3σ from average were flagged. The flagged data were checked to see if they reflect a true event or they are outliers, which need to be eliminated. Since, there are no surface measurements of AOD parameters available, such as LIDAR measurements, during QA/QC procedure AOD and PM_10_ data were also evaluated with each other during the modeling period. In fact, AOD and PM_10_ values were patterned together, and days with extreme changes in each of AOD or PM_10_ values without considerable change in the other parameter were flagged as suspicions value and double checked for potentially being outliers. By applying this method about 10-15% of data were excluded. This screening process performed on the dataset to lessen the effects of potential errors in AOD measurement by satellite and PM_10_ recorded at stations. However this would not completely eliminate the potential errors in such measurements, which could impose some extent of uncertainties to the modeling’s results.

### Regression models

The first step in the modeling process is determining the model structure to predict PM_10_ concentration as the dependent variable. Several regression models were developed and compared in this research to estimate the PM_10_ concentration using the satellite measured data. These models were improved by incorporating the meteorological parameters as well as AOD data. Most of previous studies focused on the single-variable linear regression between PM_10_ concentration and AOD data measured by different satellite sensors [17,19,33-35]. The single-variable linear regression was used as the first step in this research to analysis the linear relationship between AOD and PM_10_, and assess the possibility of using AOD parameters to develop more complex models. Single-variable linear regression model is defined by Equation 4.

4 $$ \left[P{M}_{10}\right]={\alpha}_0+{\alpha}_{AOD}(AOD) $$

where [*PM*_10_] is *PM*_10_ concentration measured at the ground stations, and *α*_0_ and *α*_*AOD*_ are intercept and slope of single-variable linear models respectively. Consequently, multiple variable regression models were defined to incorporate both AOD and meteorological data. The multivariable linear regression model was developed as following equation (Equation 5):

5 $$ \left[P{M}_{10}\right]={\alpha}_0+{\alpha}_T(T)+{\alpha}_W(W)+{\alpha}_{Dir}(Dir)+{\alpha}_{RH}(RH)+{\alpha}_{AOD}(AOD)+{\alpha}_{PBLH}(PBLH) $$

Where T, W, Dir, RH, AOD, PBL are the temperature, wind speed, wind direction, relative humidity, aerosol optical depth and planetary boundary layer height parameters, respectively. *α*_0_ is intercept of general equation and *α*_*i*_*s* are the regression coefficients of the independent variables.

Since, some of meteorological parameters don’t have linear relationship with *PM*_10_ concentration at the ground level, we need to consider non-linear relationship of available parameters and investigate the ability of this kind of models. Previous researches also showed non-linear relationship of some affecting parameters with particle concentration [12,15,21]. So, a multivariable non-linear regression model was developed and its ability in predicting concentration at the ground level was evaluated. Suggested non-linear model in this study is obtained by utilizing and modifying the general form of equations, proposed by Liu et al. [15,21] to reflect the non-linear effects of different independent variables as expressed in Equation 6:

6 $$ \left[P{M}_{10}\right]=\left({e}^{\alpha_0+{\alpha}_T\left(\mathrm{T}\right)+{\alpha}_{Dir}\left(\mathrm{Dir}\right)}\right)\times \left({e}^{\alpha_{RH}(RH)}\right)\left( AO{D}^{\alpha_{AOD}}\right)\left( PBL{H}^{\alpha_{PBLH}}\right)\times \left({W}^{\alpha_W}\right) $$

This equation incorporates physical interoperations of relation between meteorological parameters and particulate properties into the model as extensively described in previous studies [15,21]. In Equation 6 it is assumed that vertical profile of aerosol is smooth and its concentration at different altitudes is correlated to the ground level PM concentration [12]. Due to non-linear growth of particle size with increasing of relative humidity, exponential function of RH was used as well [21].

Wind speed affects PM levels by pushing out and decreasing the pollutants levels or allowing their stagnation to increase PM_10_ concentration. Also atmospheric movements by wind can re-suspend and transport in or out the mineral dust particles. Wind direction plays an important role in PM_10_ concentration due to its effect in bringing aerosols from different regions to the studied area or flushing out the particles. To facilitate the model implementation, the log function on both sides of Equation 6 was utilized to obtain linear form of equation as following (Equation 7):

7 $$ Ln\left(\left[P{M}_{10}\right]\right)={\alpha}_0+{\alpha}_{\mathrm{T}}\left(\mathrm{T}\right)+{\alpha}_{\mathrm{Dir}}\left(\mathrm{Dir}\right)+{\alpha}_{RH}(RH)+{\alpha}_{AOD} Ln(AOD)+{\alpha}_{PBLH} Ln(PBLH)+{\alpha}_W\times Ln(W) $$

All the statistical analyses were performed by Statistical Analysis System (SAS) software (version 9.1). Statistical analysis performed on the data set included to fit linear and nonlinear multivariable regression models, and calculation of regression coefficients, R^2^ values and other statistics for all kind of models. In order to perform correlation analysis and check linear single-variable regression model, simple correlation was performed and Pearson correlation coefficients (r) were calculated between AOD and PM_10_. Also, for the regression analysis and fitting multiple variable regression models least square analysis was done by SAS to fit regression models.

## Results and discussion

### Data overview

Statistical overview of PM_10_, PBLH and meteorological parameters for the datasets used to develop MISR and MODIS equations are shown in Table 1. These statistics indicate the wide range of variations in the measured PM_10_ as well as meteorological parameters, which is rather unique to the studied region and was not the case for most of the previous similar studies [15,19,21,36]. These circumstances helped to assess the ability of the developed model to compute the extreme PM_10_ concentrations.

**Table 1:** **Statistical overview of PM**_**10**_ **and meteorological measurements during year 2009**

**Parameter**	**PM**_**10, MODIS**_ $$ \left(\raisebox{1ex}{$\boldsymbol{\mu} \boldsymbol{g}$}\!\left/ \!\raisebox{-1ex}{${\mathbf{m}}^{\mathbf{3}}$}\right.\right) $$	**PM**_**10, MISR**_ $$ \left(\raisebox{1ex}{$\boldsymbol{\mu} \boldsymbol{g}$}\!\left/ \!\raisebox{-1ex}{${\mathbf{m}}^{\mathbf{3}}$}\right.\right) $$	**Temp**_**MODIS**_ **(°C)**	**Wind**_**MODIS**_ **(m/s)**	**Temp**_**MISR**_ **(°C)**	**Wind**_**MISR**_ **(m/s)**	**RH (%)**	**PBLH (m)**
Average	72.27	76.70	21.04	4.41	21.04	4.09	38.42	1173.05
STDEV	64.19	62.68	9.97	2.48	9.67	2.48	16.13	879.07
MIN	4.51	14.62	0.58	0.00	0.25	0.00	9.50	98.60
MAX	962.31	947.40	41.00	15.44	40.50	12.95	87.00	4000

The parameters with MODIS and MISR subscripts were extracted for the time that corresponds to the satellite overpass time on the stations, respectively.

**Figure 2:**
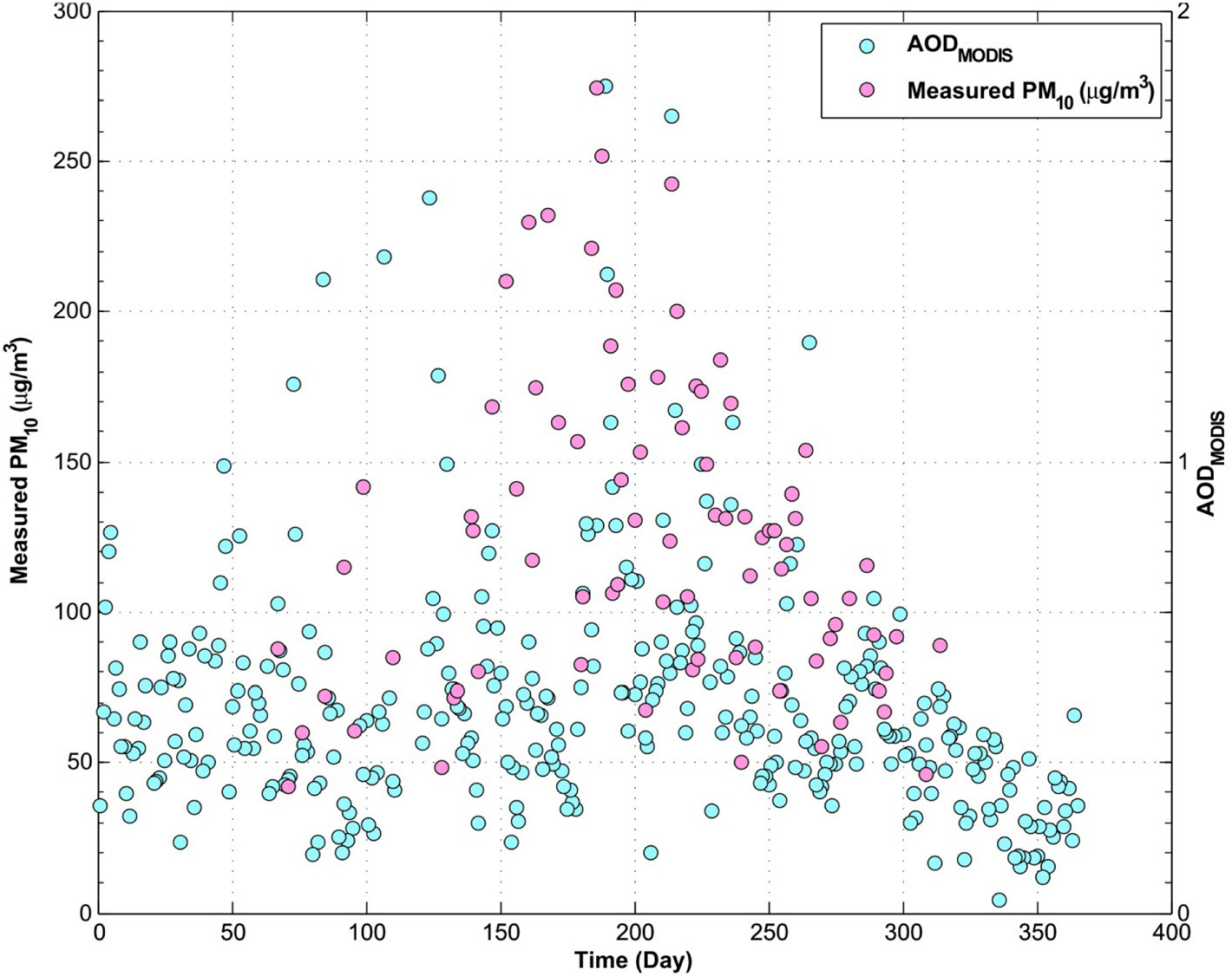
**Variation of AOD and PM**_**10**_ **concentration in 2009 for Aghdasiyeh station, MODIS sensor.**

As an example, the variations in the AOD values obtained from MODIS sensor and corresponding PM_10_ concentration throughout 2009 for one of the stations (Aghdasiyh) is presented in Figure 2. The total numbers of recorded AODs data used for this station were 81 by MODIS and 41 by MISR, throughout 2009. The values of AOD and PM_10_ concentrations mostly increased during the summer season. It reflects the regional dust generation, particulate re-suspension, and secondary particulate formation through photochemical activities during the hot summer days. Correlation coefficients of single-variable regression for other stations are presented in Table 2. Generally, AOD’s from MISR had better correlation with PM_10_ concentrations compared to MODIS data. The R^2^ between AOD from MODIS and MISR to their corresponding PM_10_ were from 0.15 to 0.43. These ranges of R^2^ values show the prospect of using AOD data to develop PM_10_ models, but it is crucial to take other parameters into account to increase estimations’ precisions.

**Table 2:** **Result of linear single-variable regression model**

**Parameter**	**R** ^**2**^			
	**Aghdasiyeh**	**Golbarg**	**Poonak**	**Shahre Rey**
AOD (MODIS)	0.15	0.16	0.15	0.16
AOD (MISR)	0.32	0.34	0.43	0.27

**Figure 3:**
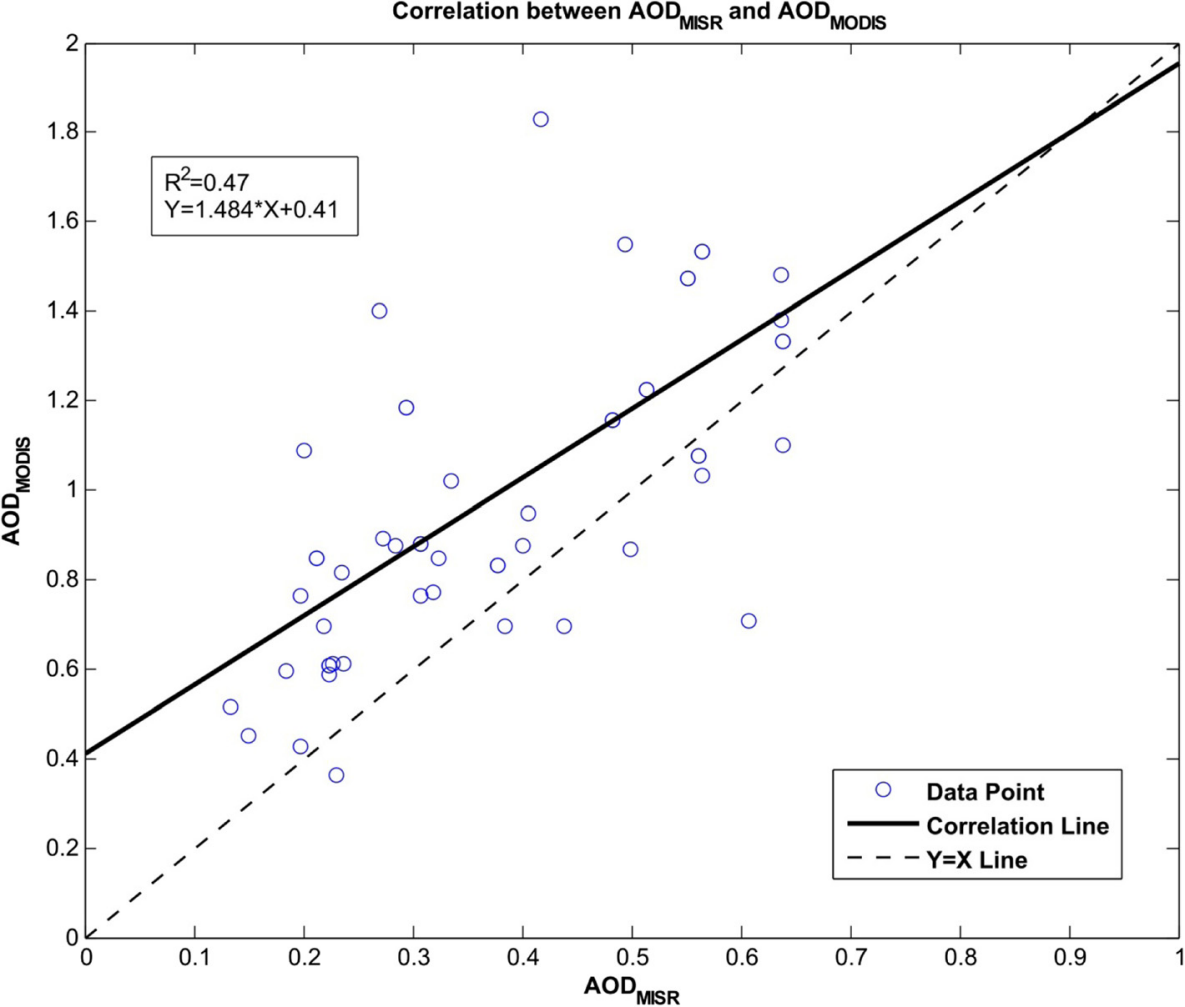
**The relationship between MISR and MODIS AOD data for all stations in 2009.**

Since ground-based measurement for the AOD parameter (e.g. an AERONET coverage) is not available for the studied region to verify the extracted satellite data and check their accuracy, AOD data from MODIS are compared to their corresponding MISR data over all the stations (Figure 3). The correlation coefficient (R^2^) between these two AOD data sets was 0.47. AOD data used in this comparison were measured in the same day by two sensors. Although these two sensors measure AOD at different time of a day, due to the proximity of the measurement time, their correlation in some extent could indicate the validity of the extracted data.

### Regression analysis

Before assessing the ability of different types of models in estimating the PM_10_, the independent variables and their coefficients are discussed in this section considering their physical meaning and atmospheric conditions in Tehran. These resulted regression coefficients are presented in Table 3. As it was expected, the coefficient sign for AOD parameter was positive in all models, which reflects the relation between PM_10_ concentrations and AOD values.

**Table 3:** **Regression coefficients for linear and non-linear multivariable regression models**

**Model type**	**Sensor**	***α***_**0**_	***α***_***AOD***_	***α***_***T***_	***α***_***W***_	***α***_***Dir***_	***α***_***RH***_	***α***_***PBLH***_	***R*** ^**2**^	${\boldsymbol{R}}_{\mathbf{Adj}}^{\mathbf{2}}$	***P − *** ***Value***	***RMSE***
Linear	MODIS	58.96	15.49	23.15	−14.36	14.52	−19.32	−0.26	0.31	0.23	0.0011	25.58
	MISR	75.20	30.72	−28.90	−13.08	5.54	−22.79	19.17	0.47	0.30	0.0400	19.71
Non-linear	MODIS	4.02	0.17	0.26	−0.19	0.18	−0.28	0.06	0.32	0.25	0.0006	0.33
	MISR	4.05	0.50	−0.36	−0.06	0.07	−0.29	0.24	0.49	0.33	0.0290	0.29

α_0_ is intercept of general equation and α_i_s are the regression coefficients of the independent variables.

In the study region anthropogenic sources are the main reason of high level of PM concentration most days in a year. In addition, dust storms from western parts of Iran increase particles concentration in the dusty episodes [28]. According to this explanation, wind could have various effects on PM concentrations; in one aspect, wind could prevent stable condition in the region and cause pollutant to flush out and dilute and spread pollutants in wider region in height and area. So, it causes PM concentration to be low in the stations. In the other hand, it could re-suspend and transport mineral dust of different size distributions to the study area and increase PM concentration at the surface. Also, due to the dust storm phenomena in our region, wind could transport large amount of dust in to the area and increase PM level in the selected stations. In our case the first situation was dominant and in most cases wind coefficient had negative sign (by increasing in wind speed value, PM concentration decrease), since data from urban stations rather close to the major pollutant sources (vehicular sources) were utilized.

High temperatures could be a sign for intensifying generation of the secondary pollutants due the photochemical activity, increasing the PM_10_ concentration at the surface level. On the other hand, the elevated PM levels could also occur during the cold periods of the year, such as during the occurrence of the inversion phenomenon in winter times. So both positive and negative coefficients were obtained for the independent variable of temperature. Negative signs were obtained for relative humidity, which represents the reverse effect of RH on AOD. Under high relative humidity condition (RH=>80%) hygroscopic particles (e.g. ammonium nitrate and ammonium sulfate) can grow into 2–10 times of their normal size, increasing the light extinction efficiencies of particle, while PM_10_ are measured at the surface stations under the controlled condition (RH=40%). Hence, the same AOD value at high relative humidity corresponds to lower particle dry mass compared to obtained value at low humidity [21].

### Multivariable regression models for individual stations

**Figure 4:**
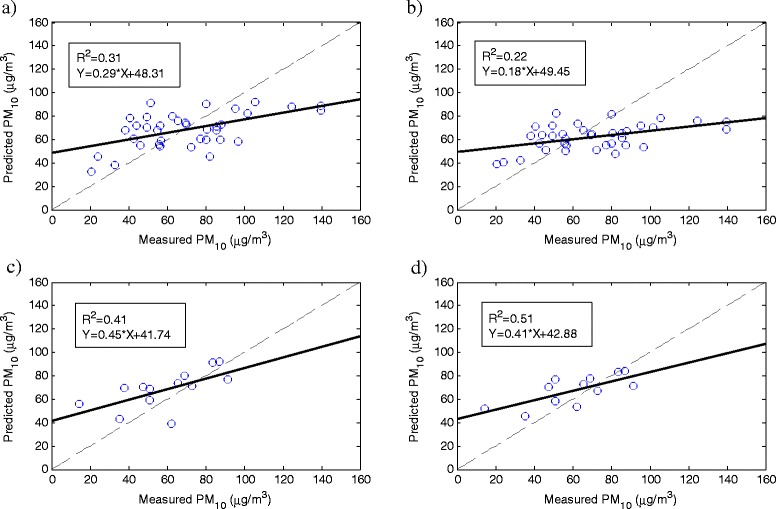
**Scatter plots of measured vs predicted PM**_**10**_ **concentration for the Aghdasiyeh station during the validation period related to a) MODIS linear model, b) MODIS non-linear model, c) MISR linear-model, d) MISR non-linear model.**

The regression parameters for both multivariable linear (Equation 5) and non-linear (Equation 6) models using AOD from either of MODIS and MISR sensors are presented in Table 3. Models obtained for MISR sensor had higher correlation coefficient and lower RMSE. Whilst MISR AODs have lower spatial resolution (17.6 Km) than MODIS AODs (10 Km), generally their corresponding model performed better. Once the models’ coefficients were adjusted using the 2009 data, the first six month of 2010 data were used to validate the obtained models. The PM_10_ levels predicted during the validation period in 2010 for Aghdasiyeh station are shown in Figure 4. Linear and Non-linear MISR models (with R^2^ of 0.41 and 0.51, respectively) showed better capability in predicting PM_10_ concentration compare to their corresponding models using AOD from MODIS. Statistical parameters, correlation coefficients between measured and modeled PM_10_, slope and intercept of validation equations during the validation period of 2010 for each station, are shown in Table 4. Whilst the number of AOD data recorded by MISR was less than those of MODIS recordings for all the stations, MISR model generally outperformed the MODIS model. Temporal resolutions of AOD data recorded for Tehran’s stations were 2–9 days for MISR and 1 day for MODIS, which resulted in less number of AOD data in MISR recordings compared to those of MODIS recordings. Linear and non-linear models obtained from MODIS data showed nearly similar performance in PM_10_ estimation except for the Aghdasiyeh station where linear model performed better. Estimations by non-linear model for MISR sensor correlated better with measured PM_10_ at ground level compared to the linear model. Among all, non-linear MISR models showed superior capability in predictions, and could estimate PM_10_ concentration with higher accuracy and less error.

**Table 4:** **Statistical Parameters for validation period of models**

**Station**	**MISR AOD**											
	**Linear model**						**Non-linear model**					
	**R** ^**2**^	**Slope**	**Intercept**	**RMSE**	**MAE**	**Bias**	**R** ^**2**^	**Slope**	**Intercept**	**RMSE**	**MAE**	**Bias**
Aghdasiyeh	0.31	0.29	48.3	23.9	19.8	2	0.23	0.18	49.5	26.6	21.1	8.4
Golbarg	0.16	0.14	55.7	25.1	20	7.7	0.14	0.1	56.2	26.4	21.1	10.4
Poonak	0.22	0.26	44.6	15.9	13.8	0.2	0.23	0.25	44.3	15.7	13.5	1.1
Shahr Rey	0.25	0.34	53.5	28.9	26.8	20.9	0.25	0.32	51.6	26.8	24.5	18.2
**Station**	**MISR AOD**											
	**Linear model**						**Non-linear model**					
	**R** ^**2**^	**Slope**	**Intercept**	**RMSE**	**MAE**	**Bias**	**R** ^**2**^	**Slope**	**Intercept**	**RMSE**	**MAE**	**Bias**
Aghdasiyeh	0.41	0.45	41.7	19.2	15.5	9.4	0.51	0.41	42.9	16.8	13.1	6.6
Golbarg	0.3	0.51	35.1	22	17.4	5.2	0.35	0.39	41.7	20	16.5	4.4
Poonak	0.50	0.89	11.9	17.9	16.2	6.3	0.55	0.65	25.1	14.7	13	6.8
Shahr Rey	0.17	0.41	60.1	36.3	34.1	25.2	0.30	0.64	46.4	32.9	30.1	26.1

It could be inferred from MODIS sensor validation results, shown in Table 4, the developed models performed rather similarly in predicting PM_10_ concentration over all four stations except for Golbarg station. Although, models in Shahr Rey have a moderate R^2^ (0.25) but its RMSE, MAE and bias (the difference between average of measured and predicted concentrations) show high values of error in predicted concentrations. On the other hand, models developed by data from MISR sensor showed weaker performance at Golbarg and Shahr Rey stations (low R^2^ and high error). Since these stations are located in the regions near the central desert of Iran and satellite sensors encounter more uncertainties in AOD retrieval from bright surface. These uncertainties could diminish models accuracy in such stations.

During several episodes of each year the Middle Eastern dust intrusions impact a vast area including the city of Tehran. During these episodes large volume of particles entrain to atmosphere, and transport several kilometers downwind, and substantially increase the PM levels in these areas. Hence, it is crucial to evaluate the ability of models to estimate high PM levels during these dust intrusion episodes. During the studied period in 2010, dust intrusions occurred in episodes of May, 18^th^-19^th^ and June 22^nd^-23^rd^. Result for measured and predicted PM_10_ by MODIS and MISR sensors during these two episodes are presented in Table 5. By comparing the predicted values with the measured PM_10_ concentrations non-linear model performed slightly better in predicting PM_10_ concentration. Generally, the MODIS models resulted in more precise prediction of PM_10_ during the dust episodes compared to the MISR models. However, the PM_10_ levels were not predicted for some days of these episodes since the AOD data were not recorded. The variations of the modeled PM_10_ generally covered a narrower range than the actual levels. Consequently, the ability of the constructed models decreases while predicting high PM_10_ levels, including the dust episodes’ levels. The results of this section indicate the ability of using AOD data to predict ground-level PM_10_, with a moderate precision.

**Table 5:** **Estimating PM**_**10**_ **concentration during the dust episodes using AODs from MODIS and MISR sensors**

**Station**	**MODIS sensor**						**MISR sensor**					
	**18 May**			**22 Jun**			**18 May**			**22 Jun**		
	**Measured** $$ \left(\raisebox{1ex}{$\boldsymbol{\mu} \boldsymbol{g}$}\!\left/ \!\raisebox{-1ex}{${\mathbf{m}}^{\mathbf{3}}$}\right.\right) $$	**Predicted** $$ \left(\raisebox{1ex}{$\boldsymbol{\mu} \boldsymbol{g}$}\!\left/ \!\raisebox{-1ex}{${\mathbf{m}}^{\mathbf{3}}$}\right.\right) $$		**Measured** $$ \left(\raisebox{1ex}{$\boldsymbol{\mu} \boldsymbol{g}$}\!\left/ \!\raisebox{-1ex}{${\mathbf{m}}^{\mathbf{3}}$}\right.\right) $$	**Predicted** $$ \left(\raisebox{1ex}{$\boldsymbol{\mu} \boldsymbol{g}$}\!\left/ \!\raisebox{-1ex}{${\mathbf{m}}^{\mathbf{3}}$}\right.\right) $$		**Measured** $$ \left(\raisebox{1ex}{$\boldsymbol{\mu} \boldsymbol{g}$}\!\left/ \!\raisebox{-1ex}{${\mathbf{m}}^{\mathbf{3}}$}\right.\right) $$	**Predicted** $$ \left(\raisebox{1ex}{$\boldsymbol{\mu} \boldsymbol{g}$}\!\left/ \!\raisebox{-1ex}{${\mathbf{m}}^{\mathbf{3}}$}\right.\right) $$		**Measured** $$ \left(\raisebox{1ex}{$\boldsymbol{\mu} \boldsymbol{g}$}\!\left/ \!\raisebox{-1ex}{${\mathbf{m}}^{\mathbf{3}}$}\right.\right) $$	**Predicted** $$ \left(\raisebox{1ex}{$\boldsymbol{\mu} \boldsymbol{g}$}\!\left/ \!\raisebox{-1ex}{${\mathbf{m}}^{\mathbf{3}}$}\right.\right) $$	
		**Linear**	**Non-linear**		**Linear**	**Non-linear**		**Linear**	**Non-linear**		**Linear**	**Non-linear**
Aghdasiyeh	55.80	56.30	56.50	80.90	69.00	65.80	62.40	39.10	53.20	93.60	91.10	83.00
Golbarg	61.50	-	-	81.30	67.60	68.70	61.20	28.90	44.90	80.80	98.10	84.60
Poonak	57.20	55.76	57.8	62.10	71.16	70.40	44.80	17.50	40.10	65.70	89.00	74.30
Shahr Rey	79.40	66.55	67.8	40.80	72.91	67.30	84.00	64.80	71.30	70.00	110.90	102.30

### Multivariable regression models over all the stations

**Figure 5:**
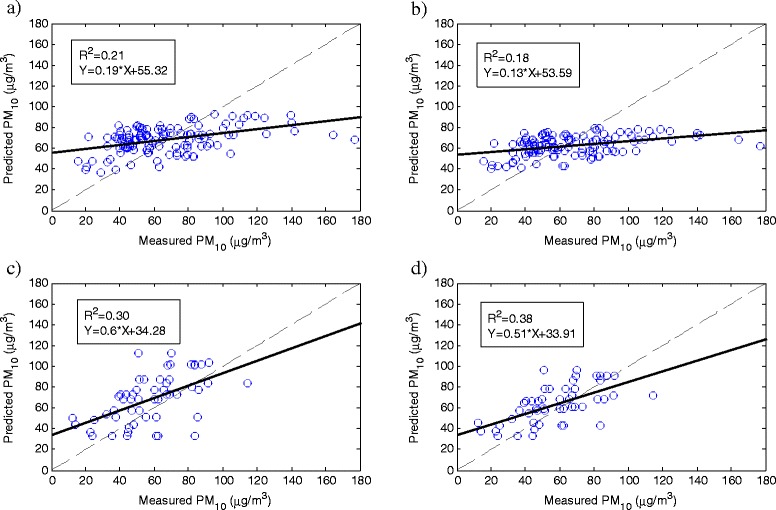
**Scatter plots of measured vs predicted PM**_**10**_ **concentration for the all stations data during the validation period related to a) MODIS linear model, b) MODIS non-linear model, c) MISR linear model, d) MISR non-linear model.**

So far, the individual models were obtained and evaluated for each individual station. However, it is crucial to examine the possibility of developing a single model over all the stations and evaluate its capability in estimating PM_10_. The values regarding the performance of the developed model over all the stations using AODs from each of MODIS and MISR are shown in Table 6. Estimations during the validation period for the linear and non-linear regression models are presented in Figure 5. All statistical parameters for developed model in validation period are shown in Table 7. Linear MISR models with R^2^ of 0.30 performed better than linear MODIS models in predicting PM_10_ concentration. This MISR sensor’s outperformance recurs for non-linear regression model with R^2^ of 0.38. By comparing other statistical parameters for all four types of the developed models it could be seen that higher values of R^2^ correspond to lower value of RMSE, MAE and bias. In fact, all statistical parameters resulted in similar drawn conclusion in evaluating models performance.

**Table 6:** **Statistical coefficients obtained from all the data**

**Statistical parameter**	**MODIS sensor**		**MISR sensor**	
	**Linear**	**Non-Linear**	**Linear**	**Non-Linear**
*R* ^2^	0.24	0.22	0.37	0.34
${R}_{\mathrm{Adj}}^2$	0.21	0.20	0.34	0.30
*RMSE*	22.62	0.32	21.70	0.32
*P* − *Value*	<0.0001	<0.0001	<0.0001	<0.0001

**Table 7:** **Statistical Parameters for validation period for models were developed in all stations**

**Type of model**	**MODIS sensor**						**MISR sensor**					
	**R** ^**2**^	**Slope**	**Intercept**	**RMSE**	**MAE**	**Bias**	**R** ^**2**^	**Slope**	**Intercept**	**RMSE**	**MAE**	**Bias**
Linear model	0.21	0.19	55.3	26.5	21.3	1.3	0.3	0.6	34.3	23.7	19.8	11
Non-linear model	0.18	0.13	53.6	27.5	21	4.3	0.38	0.51	33.9	18.5	15.1	5.9

From these R^2^ values and other statistical parameters it can be found that the ability of a single model to estimate PM_10_ concentrations at the ground surface is fair and rather lower than the individual models for the stations as expected. On the other hand the quantity of the data used to develop such models is higher than the individual models obtained for each station which make them more reliable. The ability of the single models obtained for all the studied stations to estimate the PM_10_ at 7 other stations, not included in adjusting the model parameters, were examined. An example of modeled PM_10_ data set was compared to the corresponding measured levels at these stations in Table 8. The spatial variations of the estimated PM_10_ concentrations are much lower than the actual variations. In fact, the models can’t reflect the spatial variation of concentration in the scale of the studied region. Whereas, the estimates obtained by the individual models for each station were closer to the measurements, and the range of spatial variation in the estimates were wider, and closer to the measurements.

**Table 8:** **Comparison between measured, and estimated levels by model obtained over all the stations for the new stations used for validation**

**Station**	**MODIS sensor**			**MISR sensor**		
	**Measured** $$ \left(\raisebox{1ex}{$\boldsymbol{\mu} \boldsymbol{g}$}\!\left/ \!\raisebox{-1ex}{${\boldsymbol{m}}^{\mathbf{3}}$}\right.\right) $$	**Predicted** $$ \left(\raisebox{1ex}{$\boldsymbol{\mu} \boldsymbol{g}$}\!\left/ \!\raisebox{-1ex}{${\boldsymbol{m}}^{\boldsymbol{3}}$}\right.\right) $$		**Measured** $$ \left(\raisebox{1ex}{$\boldsymbol{\mu} \boldsymbol{g}$}\!\left/ \!\raisebox{-1ex}{${\boldsymbol{m}}^3$}\right.\right) $$	**Predicted** $$ \left(\raisebox{1ex}{$\boldsymbol{\mu} \boldsymbol{g}$}\!\left/ \!\raisebox{-1ex}{${\boldsymbol{m}}^{\boldsymbol{3}}$}\right.\right) $$	
		**Linear**	**Non-linear**		**Linear**	**Non-linear**
Geophysic	57.97	60.21	59.86	60.98	36.62	47.00
Park Roz	51.61	60.54	60.18	45.79	36.57	46.00
Ostandary	54.23	60.50	60.00	73.26	36.84	48.00
Shahrdari 4	38.79	63.80	62.49	38.98	37.10	48.56
Shahrdari 11	59.77	60.50	60.20	70.90	36.24	46.65
Shahrdari 16	65.70	60.34	60.11	69.28	36.00	47.00
Shahrdari 19	84.27	60.38	60.14	87.99	36.04	46.36

The results obtained in this study were rather in similar ranges of previous studies for other regions. Models develop by Liu et al. [21] using AOD data from MISR sensor were able to estimate PM_2.5_ concentration by correlation coefficient (R^2^) of 48% in the eastern United States during the period of 2001. They continue their studies in 2007 and developed a linear regression model to predict PM_2.5_ concentration by using AOD data from MODIS and MISR with R^2^ of 51% and 62% respectively [15]. Tian et al. [12] developed a semi-empirical model by considering AOD, meteorological and boundary layer height to improve model ability. Finally, their model could explain 65% of PM_2.5_ concentration variability at the ground surface [12]. In the current study we attempted to incorporate other parameters to improve the accuracy of modeling PM_10_ by AOD data. Several models based on data from each station and total data were developed, which could acquire up to 55% explanation of PM_10_ concentrations variability by non-linear model constructed with MISR AOD data. It should be noted that most of previous studies were performed on PM_2.5_, which generally correlates better with AOD compared to PM_10_. Until now there wasn’t any relevant study in the studied region, so these results could be a good basis for the future investigation to use remote sensing data to estimate ground-level PM.

## Conclusions

Several statistical models utilizing satellite based measurements were developed and their capabilities in predicting PM_10_ concentration at the ground surface were evaluated. Linear and non-linear multi-regression models were constructed to incorporate meteorological parameters in addition to MODIS and MISR AOD data. These models were examined for stations in Tehran, Iran. Despite the significance of airborne particulate problems and the need for examining new measurement techniques, no such studies had been conducted in this area. The possibility of developing a single model over all the stations was examined and its results were compared to the individual models for each station. Performances of the constructed models to estimate PM_10_ levels during the Middle Eastern dust intrusions were examined.

In general, results of MISR models had better correlation with ground-level PM_10_ concentration compared to those of MODIS models. Non-linear multi-regression models also generally outperformed the linear models. Among all the constructed models, non-linear multi-regression models utilizing MISR AOD data resulted in the best estimates of ground-level PM_10_ ( R^2^ of up to 0.55). Generally, the models didn’t competently reflect wide temporal concentration variations, particularly due to the elevated levels during the dust episodes. However, like other periods, non-linear models performed slightly better than linear models during the dust episodes. Applying a single model over all the stations depreciate the results, while non-linear MISR model repeatedly showed the best performance being able to explain up to 38% of the PM_10_ variability. These models defined by using all data were not able to reflect the spatial variations of concentrations in the studied area. Overall, using non-linear multi-regression model incorporating both remote sensing and meteorological parameters showed a prospective in estimating ground-level PM for the studied area. However, more studies by applying other statistical models and utilizing more parameters are required to increase the model accuracies.

## Abbreviations

PM: Particulate Matter
MODIS: Moderate Resolution Imaging Spectroradiometer
MISR: Multiangle Imaging SpectroRadiometer
AOD: Aerosol Optical Depth
AERONET: AErosol RObotic NETwork
OMI: Ozone Monitoring Instrument
AQCC: Air Quality Control Company
TEOM: Tapered Element Oscillating Microbalance
RH: Relative Humidity
PBLH: Planetary Boundary Layer’s Height
GDAS: Global Data Assimilation System
